# Bacterial community shifts in response to arsenic and cadmium contamination in aquatic ecosystems: a microcosm study

**DOI:** 10.1038/s41598-026-57301-y

**Published:** 2026-06-09

**Authors:** Hayoung Lee, Mingyeong Kang, Seonah Jeong, Min-Seong Kim, Ve Van Le, So-Ra Ko, Won-Suk Choi, Dong-Yun Choi, Chi-Yong Ahn

**Affiliations:** 1 Cell Factory Research Center, Korea Research Institute of Bioscience and Biotechnology (KRIBB), 125 Gwahak-ro, Yuseong-gu, Daejeon, 34141 Republic of Korea; 2 Department of Environmental Biotechnology, KRIBB School of Biotechnology, University of Science and Technology (UST), 217 Gajeong-ro, Yuseong-gu, Daejeon, 34113 Republic of Korea

**Keywords:** Heavy metals, Freshwater, Bacterial community, Arsenic, Cadmium, Bioindicator, Ecology, Ecology, Environmental sciences, Microbiology

## Abstract

Industrialization and urbanization have led to increased levels of heavy metal pollution in aquatic ecosystems. Heavy metals exhibit toxicity even at low concentrations, posing risks to aquatic organisms, human health, and overall ecosystem health. To investigate bacterial community responses to arsenic (As) and cadmium (Cd) contamination, a microcosm experiment was conducted, followed by 16S rRNA gene amplicon sequencing, and indicator species were identified for each metal. Two concentrations (1 mg L^− 1^ and 10 mg L^− 1^) of As and Cd were applied separately, and their effects were examined. Bacterial communities in water without sediment changed more rapidly than those with sediment. In contrast, the bacterial communities in sediment and in water containing sediment remained relatively stable. Additionally, bacterial communities responded more sensitively to Cd exposure than to As at equivalent concentrations. A total of two (*Rhizorhapis* and *Methylotenera*) and six (*Polynucleobacter*, *Aquabacterium*, *Curvibacter*, *Ramlibacter*, *Methylophilus*, and *Undibacterium*) genera were identified as indicator species for As and Cd contamination, respectively, suggesting that shifts in bacterial communities can reflect contamination by specific heavy metals. These findings highlight the potential of microbial community profiling as an effective tool for the early detection and monitoring of heavy metal pollution in aquatic environments.

**Supplementary Information:**

The online version contains supplementary material available at 10.1038/s41598-026-57301-y.

## Introduction

Freshwater resources, such as rivers, reservoirs, and lakes, serve as essential water supplies and support various functions, including irrigation and power generation. These water sources also have a profound and immediate impact on human health and aquatic organisms. Rapid industrialization has introduced anthropogenic pollutants into freshwater systems. Among these pollutants, heavy metals are particularly harmful contaminants that enter rivers through various ways, such as mine discharges and industrial effluents^1,2^.

Heavy metals released by human activities pose significant threats to both human health and aquatic ecosystems^3^. Even at relatively low concentrations, they are toxic to aquatic organisms. While some metal elements are essential micronutrients required for metabolic processes, excess amounts can accumulate in human and animal tissues^4,5^. Accumulated heavy metals in aquatic organisms can further transfer to higher trophic levels through bioaccumulation, leading to serious health risks for consumers of contaminated water and aquatic organisms. This bioaccumulation ultimately threatens ecosystem stability^6,7^.

Heavy metals are critical environmental pollutants with the potential to cause neurotoxic effects. Prolonged exposure to certain heavy metals can lead to their accumulation in the developing brain. Among these, arsenic (As) and cadmium (Cd) have been extensively studied and identified as significant risk factors for human health^8,9^. Cd is commonly related to water pollution^10^ and is known to cause nausea, excessive saliva secretion, destruction of red blood cells, muscle spasms, and severe damage to the kidneys and bones through bioaccumulation in the human body^4^. Arsenic is toxic to most multicellular organisms and is classified as a carcinogen for humans^9^.

According to Pandey et al.^5^, five heavy metals (Cr, Ni, Cu, Pb, and Zn) are commonly found in water bodies, while seven (As, Cr, Ni, Cu, Hg, Pb, Cd, and Zn) are detected in sediment. In our previous measurements, As and Cd were detected in sediments from a river near the Seokpo Smelter, located in the upper reaches of the Nakdong River, an area widely recognized for heavy metal contamination. Sediments tend to accumulate higher concentrations of heavy metals than water, suggesting that these contaminants in sediment can be transported downstream through water flow^11^.

Various bioindicators have been employed to assess the ecological impact of pollution on aquatic ecosystems. However, traditional bioindicators, including macroinvertebrates, algae, and fish, present several limitations. The collection and processing of biological specimens is often labor-intensive and costly, and variability in sampling protocols or researcher expertise can introduce inconsistencies in the results^12^. These constraints highlight the need for simpler and more efficient monitoring approaches.

Microorganisms offer a promising alternative due to their ease of sampling, rapid responses to environmental changes, and broad applicability across diverse aquatic environments. Although numerous studies have demonstrated that heavy metals significantly alter microbial community composition and functional traits^13,14^, relatively limited attention has been given to identifying metal-specific bacterial taxa that can serve as practical bioindicators based on 16S rRNA gene sequencing data. Given the increasing accessibility and cost-effectiveness of high-throughput 16S sequencing, taxonomic profiling provides a powerful framework for environmental monitoring. In particular, 16S rRNA-based analyses enable the simultaneous characterization of microbial responses to multiple metal stressors within a single dataset, thereby supporting efficient multi-contaminant assessment.

Moreover, microbial communities can be utilized for both short-term and long-term ecological monitoring. Recent advancements in next-generation sequencing technologies have enabled detailed environmental microbiome analysis, revealing that bacterial communities exhibit stable seasonal succession patterns and high resilience to short-term environmental fluctuations^15–17^. Nonetheless, microbial communities can respond rapidly to contamination events such as rainfall-driven sewage overflows and fecal pollution^18^. Owing to their high sensitivity to environmental stressors, microorganisms are more responsive to heavy metal pollution than macroorganisms, making their community structure and activity valuable bioindicators for evaluating soil and water quality^19,20^. Considering these microbial characteristics, it seems appropriate to use bacteria as bioindicators for aquatic ecosystems. Investigating the disturbances in microbial communities caused by anthropogenic pollutants provides critical insights into ecosystem health^21^.

The aims of this study are (1) to investigate changes in microbial community structure in response to As and Cd exposure, (2) to identify alterations in microbial communities at different concentrations of these heavy metals, (3) to compare microbial community changes in the presence and absence of sediment, and (4) to evaluate the feasibility of using microbial communities as indicators for heavy metal contamination.

## Results

### Effect of As and Cd on bacterial compositional changes

**Fig. 1:**
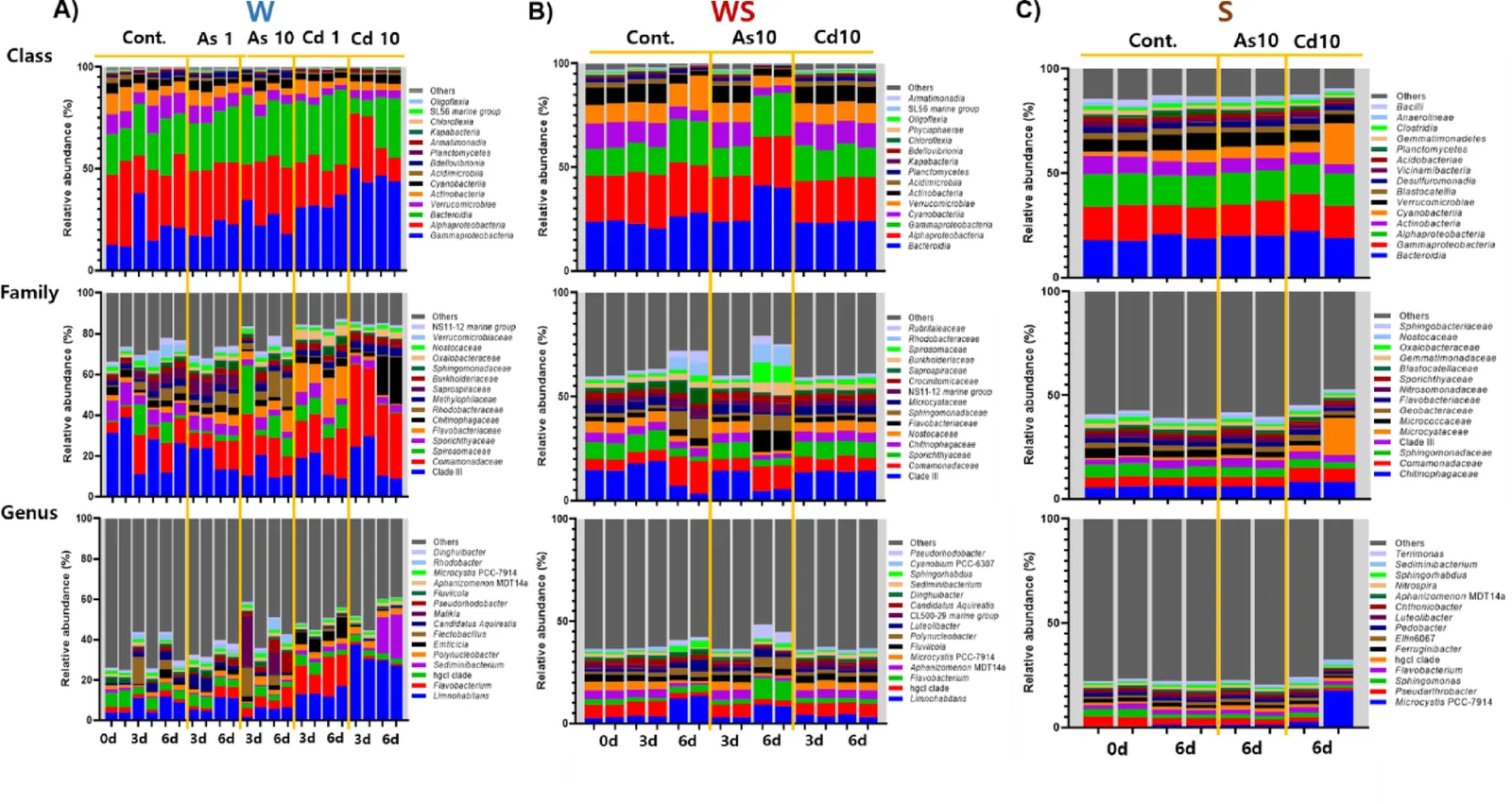
Bacterial compositional changes in response to As and Cd treatments. The graphs display the 15 most abundant taxa in each treatment group: W (**A**), WS (**B**), and S (**C**). Taxonomic composition is presented at the class, family, and genus levels. Relative abundances reflect changes over time and across different metal concentrations. Figure 1 was created using GraphPad Prism 9.

The microbial relative abundance varied depending on heavy metal concentration and exposure duration across the experimental groups (W, WS, and S) (Fig. 1). Overall, Cd altered bacterial community more strongly, compared with As, particularly at a higher concentration in the W group. These compositional changes were more evident at the genus level. In the W group, *Pseudorhodobacter* and *Malikia* increased with higher concentrations of As (Fig. 1A). In the Cd-treated groups, *Flavobacterium* and *Emticicia* were relatively more abundant at 1 mg L^− 1^, whereas *Limnohabitans* and *Sediminibacterium* became dominant at 10 mg L^− 1^.

In contrast, bacterial compositional changes in the WS group were considerably smaller than those in the W group (Fig. 1B). Following Cd treatment, no significant changes in relative abundance were observed throughout the experiment. However, in the As-treated WS group, an increase in *Flavobacterium* was detected over time. In the S group, only *Microcystis* increased after 6 days of Cd exposure (Fig. 1C). Notably, in the W group, distinct difference in microbial abundance emerged after just 3 days of metal exposure. However, the groups with sediment (WS and S) exhibited noticeable changes only after 6 days. The impact of heavy metal exposure to bacterial communities was observed more rapidly in microcosm water than in environments containing sediment.

### Comparative effect of As and Cd on temporal dynamics of bacterial communities

**Fig. 2:**
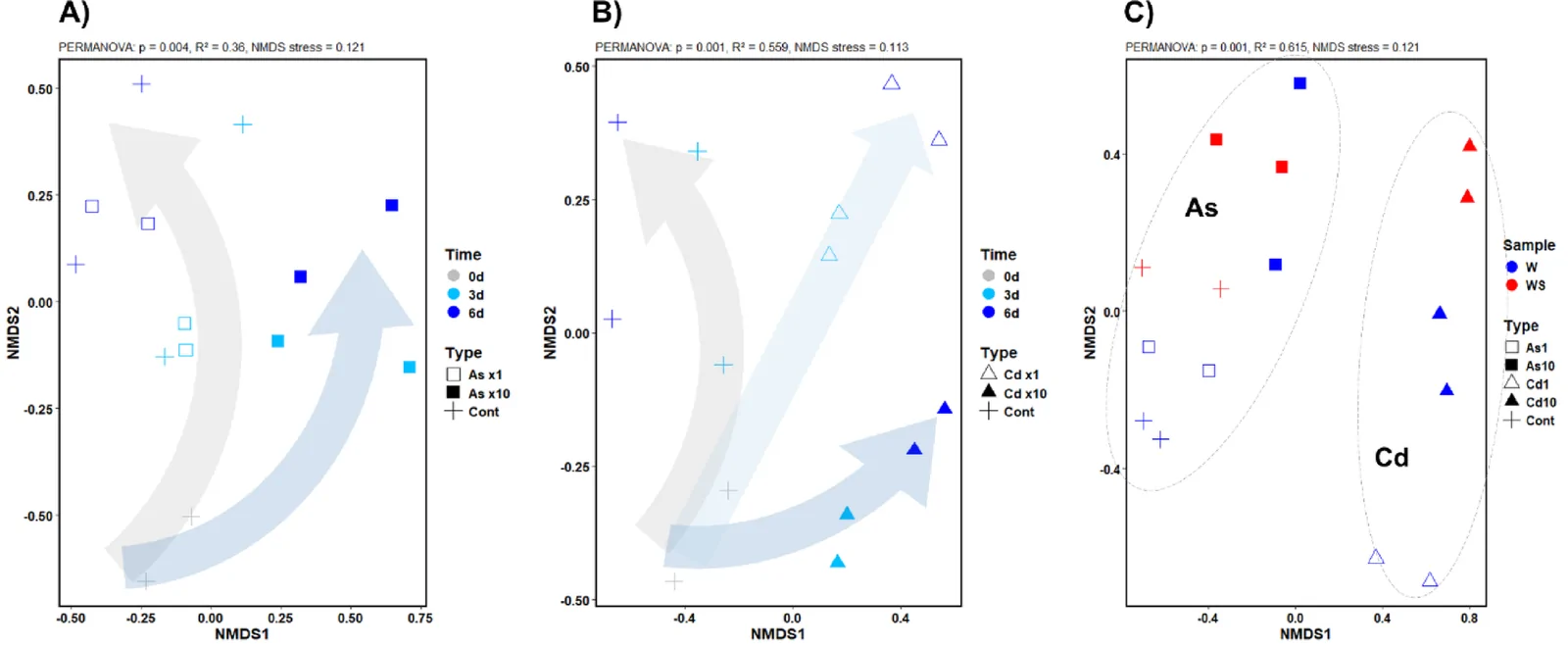
Non-metric multidimensional scaling (NMDS) plots for bacterial community changes in response to As and Cd treatments. NMDS plots illustrate the shifts in microbial β-diversity over time for each heavy metal: As (**A**) and Cd (**B**). Comparison of bacterial community composition between As- and Cd-treated samples after 6 days of exposure (**C**). Arrows (**A** and **B**) indicate the direction of bacterial community shifts during the experiment, and ellipses (**C**) denote clustering patterns differentiating the As and Cd treatments. Plots were generated using R (ggplot2 package).

A bacterial β-diversity analysis was performed to investigate temporal shifts in bacterial communities in microcosm water. It showed distinct changes in bacterial communities between treatment groups over time (Fig. 2). In As-treated samples, the control and the 1 mg L^− 1^ As treatment group exhibited similar patterns of community change, whereas the 10 mg L^− 1^ As treatment group had distinct patterns (Fig. 2A). However, the bacterial communities in Cd-treated water changed differently across all concentrations (Fig. 2B). Even at a low concentration, Cd treatment caused substantial deviations in bacterial community composition compared to the control, a pattern not observed with As treatment.

In addition, the β-diversity of the bacterial communities between As and Cd treatment, including both W and WS groups after 6 days, revealed distinct clustering (Fig. 2C). The bacterial communities exposed to As and Cd formed separate clusters, with the low-concentration As treatment group clustering closely with the control group. In general, As-treated groups were more similar to the control group, whereas Cd treatment showed more pronounced divergence from the control regardless of concentrations. When exposed to the same concentrations of As and Cd, the greater changes in bacterial community were induced for Cd. There were no significant differences in α-diversity among treatment groups or over time (Fig. S1).

**Fig. 3:**
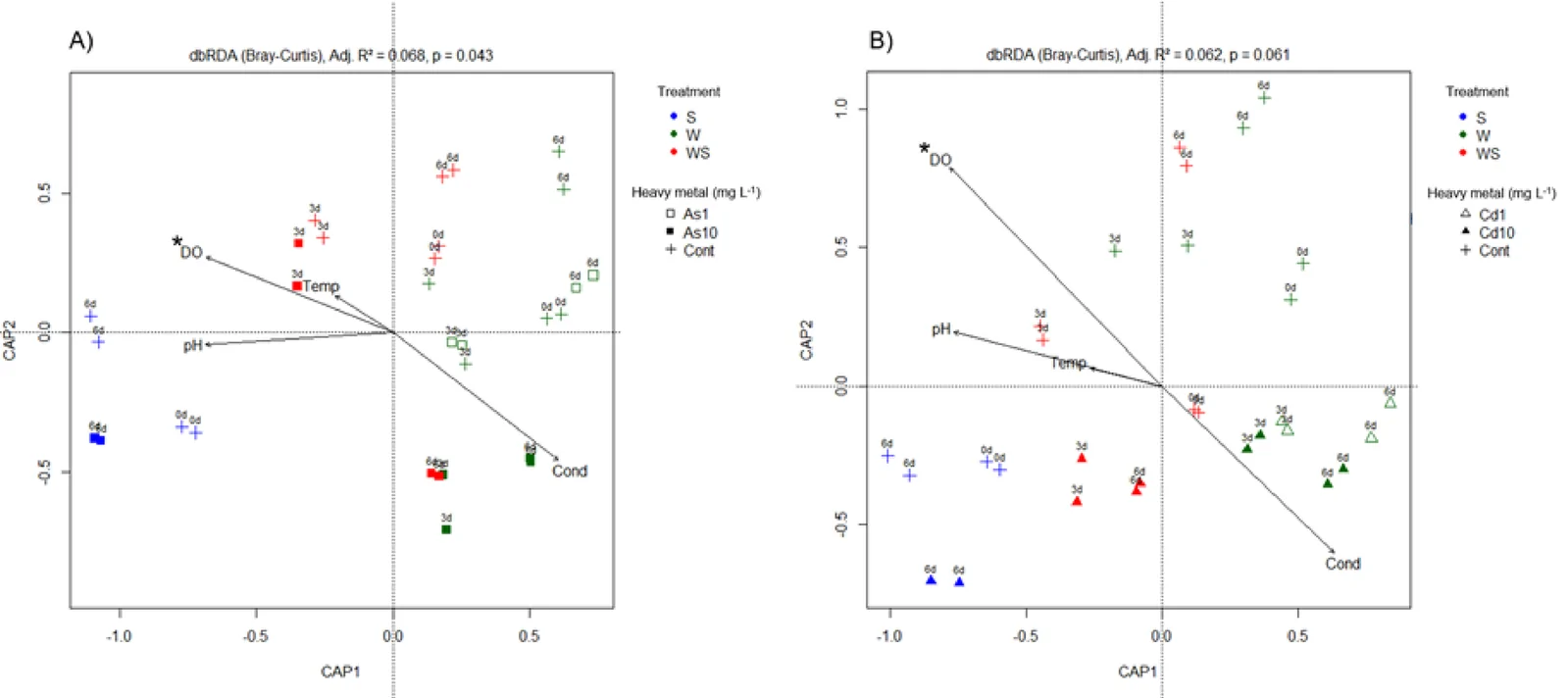
Distance-based redundancy analysis (dbRDA) of microbial communitiy composition in relation to environmental parameters under As (**A**) and Cd (**B**) treatments. Physicochemical parameters included in the analysis were measured on days 0, 3, and 6. Statistical significance is indicated as *p < 0.05, **p< 0.01, ***p < 0.001. Plots were generated using ggplot2 package in R.

The relationships among bacterial communities, the presence or absence of sediment, and environmental factors were analyzed using dbRDA (Fig. 3). The physicochemical parameter data used in the analysis are presented in Tables S1–S3. Higher heavy metal concentrations exerted stronger effects on bacterial community composition, as observed in the W treatment group. The addition of sediment shifted the aquatic bacterial communities to become more similar to those of the sediment communities. Overall, the bacterial community in the W groups was relatively associated with conductivity. This pattern was consistently observed across both metal treatments. In addition, the As 1 mg L^− 1^ treatment clustered closely with the control, which was consistent with the NMDS results.

### Bacterial bioindicators for As and Cd

When a specific heavy metal is introduced, the change in the bacterial community can be attributed to the higher survival of bacteria with stronger resistance to the heavy metal. To identify bacterial indicators for heavy metals, groups treated with As and Cd, as well as control, were analyzed at the ASV level, using indicator species analysis. The bacterial indicators were identified based on their increasing relative abundance over time and with higher heavy metal concentrations.

**Fig. 4:**
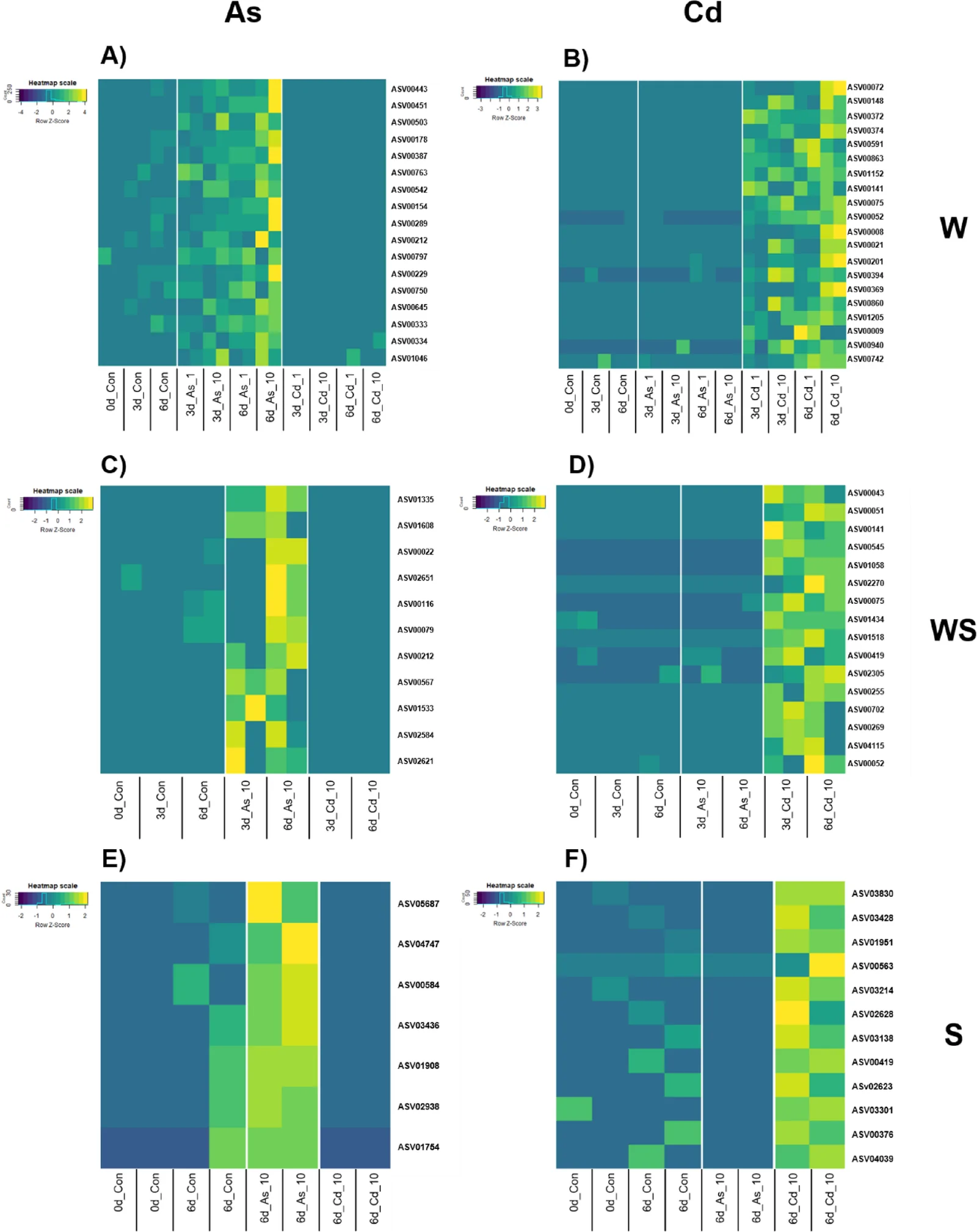
Heatmap visualization of bacterial indicator species indentified under As and Cd treatments. Heatmaps show indicators for As in W (A), WS (C), and S (E); and for Cd in W (B), WS (D), and S (F). Color intensity reflects the relative abundance of each indicator ASV. Heatmap was generated using heatmap.2 from the gplots package in R.

ASVs with higher stat values (> 0.8) and lower *P* values (< 0.05) were identified for As and Cd treatment groups. A total of 17 and 20 ASVs were selected as indicators from W groups treated with As and Cd, respectively (Fig. 4A,B). In the As-treated group, *Methylotenera* (ASV00212, ASV01046) and *Peredibacter* (ASV00154, ASV00451) were identified two times at the genus level (Table S4), while *Limnohabitans* (ASV00021, ASV00394, ASV00860), *Sediminibacterium* (ASV00008, ASV00072, ASV00369, ASV01205) *Methylophilus* (ASV00075, ASV00148, ASV00374) were repeatedly detected in the Cd-treated group (Table S5). For WS groups, 12 and 16 ASVs were identified for As and Cd, respectively (Fig. 4C,D). In the As-treated WS group, no genus or ASV was repeatedly identified. Among the ASVs identified with statistical significance, *Caulobacter* (ASV01335) and *Ottowia* (ASV01608) exhibited the highest stat values (1) and the lowest *P* value (0.0025), respectively (Table S6). In the Cd-treated WS group, *Undibacterium* (ASV00141, ASV00419) and *Paucibacter* (ASV01058, ASV02270) were each identified twice. For the S groups treated with As and Cd, 7 and 12 ASVs were identified as indicators, respectively (Fig. 4E,F). Among all experimental groups, the fewest number of indicators were identified in the S groups. In the As- and Cd-treated S groups, no genera or ASVs were repeatedly observed, whereas unknown bacterial genera were more frequently detected (Tables S8 and S9).

Despite variations in treatment groups (W, WS, and S), certain bacterial genera were repeatedly found as indicators for the same heavy metal across different conditions. *Flavobacterium*, *Rhizorhapis*, and *Methylotenera* were detected in both W and WS with As treatment. *Ramlibacter*, *Polynucleobacter*, *Methylophilus*, *Curvibacter*, *Limnohabitans*, and *Undibacterium* were found in both W and WS with Cd treatment. Despite most of the indicators being commonly found in microcosm water, *Aquabacterium* was identified in both W and S, and *Undibacterium* in W, WS, and S with Cd treatment.

Indicator bacteria that were found in common across two or more treatment groups were selected as bioindicators for each heavy metal at the genus level (Table 1). While *Flavobacterium* and *Limnohabitans* were also identified as potential indicators, their ubiquity in aquatic ecosystems renders them unsuitable as specific bioindicators. A greater number of indicators were identified for Cd (six) compared to As (two).

**Table 1:** The list of identified microorganisms as bioindicators for particular heavy metals.

Heavy metal	Phylum	Class	Order	Family	Genus
As	Proteobacteria	*Alphaproteobacteria*	*Sphingomonadales*	*Sphingomonadaceae*	*Rhizorhapis*
	Proteobacteria	*Gammaproteobacteria*	*Burkholderiales*	*Methylophilaceae*	*Methylotenera*
Cd	Proteobacteria	*Gammaproteobacteria*	*Burkholderiales*	*Burkholderiaceae*	*Polynucleobacter*
	Proteobacteria	*Gammaproteobacteria*	*Burkholderiales*	*Comamonadaceae*	*Aquabacterium*
	Proteobacteria	*Gammaproteobacteria*	*Burkholderiales*	*Comamonadaceae*	*Curvibacter*
	Proteobacteria	*Gammaproteobacteria*	*Burkholderiales*	*Comamonadaceae*	*Ramlibacter*
	Proteobacteria	*Gammaproteobacteria*	*Burkholderiales*	*Methylophilaceae*	*Methylophilus*
	Proteobacteria	*Gammaproteobacteria*	*Burkholderiales*	*Oxalobacteraceae*	*Undibacterium*

## Discussion

Anthropogenic activities are introducing various chemical contaminants into aquatic ecosystems, with heavy metals being one of the common environmental pollutants. Rivers and streams often serve as pathways for transporting heavy metals from urban and industrial wastewater to downstream regions^21,22^. Common sources of heavy metal pollution include fossil fuel combustion, smelting, sewage discharge, mining activities, metal chelates, and the indiscriminate use of pesticides and fertilizers. Arsenic (As), cadmium (Cd), copper (Cu), chromium (Cr), mercury (Hg), lead (Pb), and zinc (Zn) are the major heavy metal contaminants^23,24^.

Bacterial communities are highly sensitive to heavy metal exposure. In heavy metal-contaminated sites, bacterial communities exhibit reduced diversity and abundance, an increased prevalence of metal-resistant bacteria, and declines in microbial biomass and metabolic activity^24–26^. Heavy metals are generally toxic at low concentrations^22^. In particular, Cd is harmful to living organisms even at lower concentrations as low as 0.001–0.1 mg L^− 1^^27^. The World Health Organization (WHO) recommends a maximum acceptable concentration of As in drinking water of 10 µg L⁻¹^28^.

As and Cd were selected as representative heavy metals due to their widespread occurrence, regulatory importance, and contrasting toxicity mechanisms. The mobility and bioavailability of As and Cd in sediments are critical factors influencing aquatic ecosystems^29^. In addition, As and Cd differ in environmental mobility and sediment association, making them suitable model contaminants. As exhibits strong redox-dependent interactions with iron in sediments, leading to its sequestration or release under changing redox conditions^30^. In contrast, Cd is primarily immobilized through physical or chemical associations with organic matter and carbonate minerals in sediments^31^. This study focused on understanding how As and Cd influence bacterial community structure in aquatic ecosystems and to explore the potential use of metal-responsive bacterial taxa as bioindicators of heavy metal pollution.

Overall, the bacterial composition in the As treatment group showed a more similar pattern to the control, whereas the Cd treatment group exhibited a different pattern (Fig. 1). Cd treatment resulted in more pronounced shifts in bacterial community composition than As at equivalent concentrations, suggesting a stronger impact of Cd on the microbial community. Heavy metal solubility and mobility are known to increase under low pH and low DO conditions, which can enhance metal bioavailability. In this study, pH remained relatively stable (approximately 7–8) across all microcosm treatments, whereas DO levels decreased to 4–5 mg L^− 1^ in the Cd-treated groups (Tables S1, S2, and S3). Such conditions may have contributed to the comparatively stronger influence of Cd on microbial community change, compared to As. The compositional changes in bacterial communities were greater in W than in WS or S, compared with the control. Despite being a microcosm water, WS displayed smaller compositional changes than W, likely due to the inclusion of sediment. As previously mentioned, heavy metals tend to accumulate in sediment, potentially reducing their bioavailability and subsequent impact on bacterial communities in the water column. In contrast, the bacteria identified as indicators in S may possess greater resistance or tolerance to heavy metal exposure.

Jeong et al.^32^ investigated bacterial communities in two areas with different As concentrations. The bacterial compositions at the phylum level between the two areas were similar, except for a significant difference in the phylum Chloroflexi (group A, 12.1 ± 4.9%; group B, 2.9 ± 2.0%). These results are consistent with our findings, where no significant differences in bacterial composition were observed between the two As concentrations even at the class level (Fig. 1A). Although Chloroflexi was detected at low abundance in the present study, it increased in the As-treated samples compared with the control (ASV04747; Fig. 4E and Table S8).

Zhang et al.^33^ reported that the bacterial community structures remained similar across different Cd concentrations in soil samples, but notable changes were observed depending on exposure duration. Shorter exposure times (48 h) led to more pronounced changes than longer durations (30 days). However, temporal variation in the bacterial community of the microcosm sediment was less pronounced, likely reflecting the limited temporal resolution of the S groups, which were analyzed at only two time points (days 0 and 6) (Figs. 1C and 3). No noticeable changes in the bacterial community were observed in the S groups during the experiment. However, in the W and WS groups, more rapid changes in bacterial communities were evident, particularly in the absence of sediment (Fig. 1A,B). It is clear that the extent of bacterial community composition changes varies depending on the heavy metal and the presence of sediment, with Cd causing more pronounced changes than As at the same concentrations.

Changes in the bacterial community were distinct depending on the concentrations of As and Cd (Fig. 2). The bacterial community was more sensitive to Cd than to As, as the bacterial community changed more significantly from the control when treated with a low concentration of Cd, compared to As (Fig. 2A,B). Moreover, the clustering of bacterial communities differed between As- and Cd-treated groups (Fig. 2C), indicating that the bacterial communities respond differently to As and Cd, likely due to variations in the sensitivity or resistance of specific bacterial species to each heavy metal. These results suggest that the compositional changes in the bacterial community could be a useful tool for distinguishing between As and Cd contamination in aquatic environments. Differences in sensitivity or resistance among bacterial taxa highlight the potential of using bacterial communities as bioindicators for specific heavy metals.

Furthermore, changes in bacterial community composition were influenced by habitat type (water or sediment) rather than solely by metal concentration (Fig. 3). The W, WS, and S groups were associated with different environmental factors, suggesting that bacterial communities in the microcosm water and sediment compartments respond to distinct environmental conditions. Nevertheless, most of the observed variation in bacterial community composition was likely driven primarily by heavy metal exposure rather than by environmental factors alone. First, regardless of the type of heavy metal treatment, the overall patterns of environmental parameters associated with W, WS, and S groups were largely similar, and none of the measured environmental parameters, except for DO, showed statistically significant relationships with the bacterial community composition. Second, in the dbRDA analysis, the conductivity vector aligned in the opposite direction of the DO vector and both showed significant associations with the bacterial community composition in the W groups. This pattern suggests that the bacterial assemblages were associated with conditions characterized by relatively lower DO and higher conductivity. Such opposing trends between DO and conductivity may reflect redox-driven geochemical processes, as decreasing DO is known to promote the mobilization of sediment-bound metals through reductive dissolution of metal oxides, thereby increasing dissolved ion concentrations and conductivity^34,35^. Therefore, DO may act as an indirect environmental proxy reflecting underlying redox conditions rather than a sole driver of community variation. In addition, bacterial communities in water and sediment should be examined separately, as they exhibited distinct response patterns. Notably, community shifts are more pronounced in water than in sediment.

In terms of diversity, all indices showed no significant differences between treatment and control groups (Fig. S1). Although the Chao index was significantly higher in S compared to W and WS, it is likely attributed to the naturally higher diversity in sediment environments. Similar findings were reported by Kou et al.^36^, who found no significant impact of heavy metal concentrations on bacterial relative abundance and diversity across six coal gangue sites. Additionally, Jeong et al.^32^ observed no notable differences in bacterial richness and diversity at sites with varying As concentrations. These results are consistent with our findings, indicating that bacterial diversity remains relatively stable even in the presence of heavy metals, potentially due to compensatory shifts, namely, the decline of sensitive taxa alongside an increase in metal-resistant species^37^.

Indicator bacteria can serve as valuable tools for monitoring specific heavy metal contamination by examining the changes in bacterial communities in response to heavy metals. Since microorganisms capable of surviving in heavy metal-contaminated water or sediment have resistant characteristics^38,39^, selected indicator species have the potential to provide insights into the presence and levels of heavy metal contamination.

Among the indicator species found in the W group, *Methylotenera*, a methylotrophic bacterium, is reported to increase in As-contaminated waters^40^(Fig. 4A,C; Tables 1, S4, and S6). *Rhizorhapis*, identified in As-treated W and WS groups, possesses resistance genes for As, Cu, Pb, and Hg^41^(Fig. 4A,C; Tables 1, S4, and S6). *Caulobacter*, detected in As-treated WS, possesses genes associated with heavy metal resistance^42^(Fig. 4C and Table S6). Kou et al.^36^ found that Actinobacteriota and Proteobacteria are resistant to Cd. In our experiments, many indicator species identified in the Cd-exposed bacterial community belonged to the phylum Proteobacteria. Among them, *Gammaproteobacteria*, a class within Proteobacteria, showed a greater increase in Cd-treated W compared to As-treated W, whereas *Alphaproteobacteria* exhibited a decrease. This suggests that *Gammaproteobacteria* may possess greater resistance to Cd compared to other bacterial classes within Proteobacteria. *Methylophilus*, a methanol-utilizing bacterium with Cd-accumulating potential, has resistance to Cd^43,44^(Fig. 4B,D; Tables 1, S5, and S7). *Ramlibacter* and *Curvibacter* were also resistant to Cd (Fig. 4B,D; Tables 1, S5, and S7). *Ramlibacter* has been found to increase in Cd-contaminated soils^45,46^, and *Curvibacter* is dominant in environments with high levels of heavy metals^47,48^. *Undibacterium*, identified as a Cd indicator in W, WS, and S, is associated with Fe-redox cycling^47^ (Fig. 4B,D,F; Tables 1, S5, S7, and S9). Similarly, *Aquabacterium*, a nitrate-dependent Fe (Ⅱ)-oxidizing bacterium, was also detected in Cd-treated W and S groups^49^(Fig. 4B,F; Tables 1, S5, and S9).

Based on the results of this study, *Rhizorhapis* and *Methylotenera* were chosen as indicators for As contamination, while *Polynucleobacter*, *Aquabacterium*, *Curvibacter*, *Ramlibacter*, *Methylophilus*, and *Undibacterium* were selected as indicators for Cd (Fig. 4; Table 1). These genera were consistently detected in at least two of the W, WS, and S groups exposed to each metal, supporting their potential use as bioindicators of heavy metal contamination in both water columns and sediment-influenced environments. Furthermore, the fact that none of the identified indicator bacteria overlapped between the As- and Cd-treated groups underscores the metal-specificity of these bioindicators and suggests their utility in distinguishing different types of contamination. The bacterial taxa identified as potential bioindicators in this study have previously been reported to possess resistance mechanisms against heavy metals, as discussed above. Bacteria employ diverse metal-specific resistance strategies, including efflux systems, or detoxification pathways. Therefore, beyond As and Cd, other metal-responsive bacterial taxa may also be detectable using the 16S rRNA gene-based community profiling approach applied in this study. These findings suggest the broader applicability of this method for identifying candidate bacterial indicators of various heavy metal contaminants.

Under the heavy metal-enriched conditions examined here, functional predictions derived solely from 16S rRNA gene data were unlikely to provide substantial novel insight beyond the observed taxonomic responses. Future studies incorporating metagenomic or transcriptomic approaches would provide more reliable mechanistic insights.

As mentioned above, As and Cd exhibit different mechanisms of toxicity in bacteria; consequently, the observed microbial responses should be interpreted in the context of metal-specific effects. Natural aquatic ecosystems are inherently more complex than controlled microcosm systems, because multiple physicochemical and biological variables interact simultaneously. While our short-term microcosm experiments provide preliminary evidence for the potential of these bacterial taxa as bioindicators, these findings are limited to specific metal concentrations and experimental conditions employed.

The concentrations used in this study exceed typical environmental levels. However, comparable levels have also been reported in heavily contaminated urban areas^50^ and mining communities^51,52^. The experimental As and Cd concentrations were intentionally selected to enhance response detectability and to elucidate underlying mechanisms of metal-induced stress. While the magnitude of responses cannot be directly extrapolated to natural systems, the qualitative patterns observed, such as taxa-specific sensitivity, likely reflect generalizable physiological constraints. Under environmentally relevant concentrations, similar processes may occur with reduced intensity. The inclusion of sediment in our microcosms partially reflects natural ecosystem complexity, yet the applicability of these conclusions to broader environmental contexts – including variable metal levels, complex biotic interactions, and longer temporal scales – remains to be validated. Therefore, long-term field-based monitoring is necessary to confirm the ecological relevance and generalizability of these bacterial indicators under natural conditions.

## Conclusion

This study investigated the response of bacterial communities to As and Cd contamination under both water column and sediment conditions. Our findings revealed that bacterial communities responded differently depending on the type of heavy metal and environmental condition. The bacterial community exposed to Cd exhibited greater compositional changes compared to that exposed to As at equivalent metal concentrations. Additionally, the presence of sediment mitigated the extent of community change, with smaller shifts observed in sediment-containing systems than in water-only conditions. This likely reflects the tendency of heavy metals to accumulate in sediment, reducing their bioavailability in the water column, as well as inherent differences in microbial tolerance to heavy metals between sediment and planktonic communities. The bacterial indicator taxa identified for each metal exhibited traits associated with heavy metal resistance or adaptation. Given that distinct indicator taxa were associated with As and Cd, this study supports the potential use of bacterial communities as specific bioindicators for heavy metal pollution. Our findings suggest that bacterial taxa previously reported to possess heavy metal resistance may serve as promising metal-specific bioindicators for aquatic environmental monitoring. Importantly, the identification of heavy metal-responsive bacterial indicators provides a basis for the development of more practical, targeted, and sensitive biomonitoring strategies. Such microbial indicators may complement traditional macroorganism-based approaches by capturing rapid and subtle ecological responses to contamination. These results contribute to a broader understanding of microbial ecological dynamics under metal stress and highlight the potential applicability of microbial community analyses in environmental health assessment. Future studies validating these indicators in natural aquatic systems will be essential for establishing their robustness and generalizability in environmental monitoring programs.

## Materials and methods

### Microcosm setup

The sediment and water for microcosm experiments were collected on May 19, 2023, from a site in the Nakdong River, Korea (35° 43’ 30.9” N, 128° 25’ 55.3” E), where no heavy metals have been detected over the last five years (Table S10). The river bed at the sampling location is primarily covered by finer particles than sand. The water sample was filtered with a 25-µm sieve to remove suspended particles and sand. Both sediment and filtered water were immediately transported to the microcosm experimental site.

**Fig. 5:**
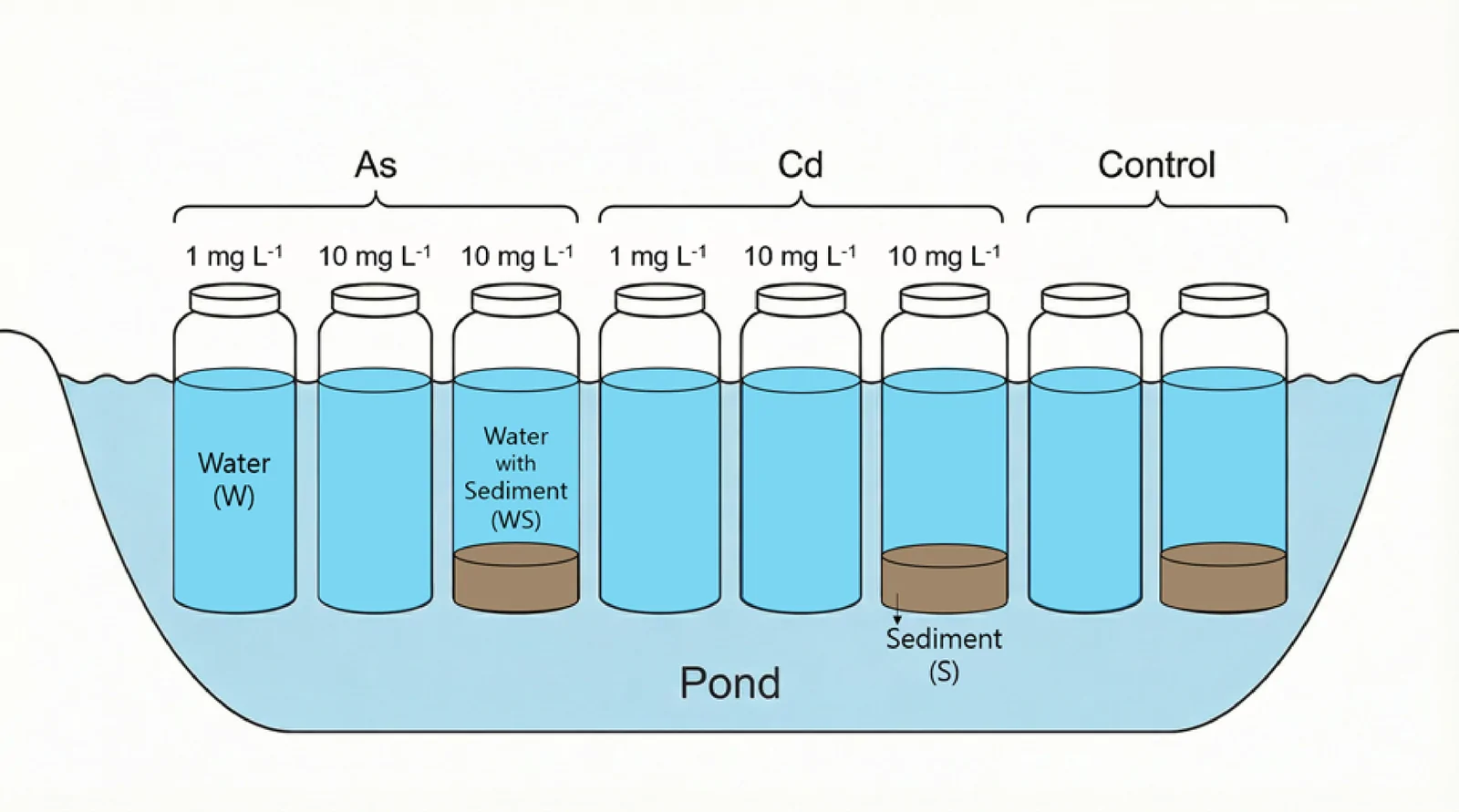
Schematic representation of the microcosm experiment setup. Two conditions were tested: (1) Water only (W) and (2) Water and sediment. Samples were collected from three components: (i) W, (ii) Water (WS) from Water and sediment, and (iii) Sediment (S) from Water and sediment. Each microcosm contained 4 L of filtered river water, with or without 800 g of sediment, and was treated with either 1 mg L^-1^ or 10 mg L^-1^ of arsenic (As) or cadmium (Cd). Control groups without metal addition were also included.

A microcosm experiment was conducted in a pond at the Korea Research Institute of Bioscience and Biotechnology. Transparent plastic bottles (10 L capacity) were filled with 4 L of filtered water. Cd and As were added separately to the bottles at concentrations of 1 mg L^− 1^ and 10 mg L^− 1^, respectively, for each metal treatment (Fig. 5). To determine the impact of heavy metals on bacterial communities within sediment, an additional microcosm was prepared by combining 4 L of filtered water and 800 g of sediment and treating it with either As or Cd at 10 mg L^− 1^. The control group for water and sediment was filled with 4 L of sample water and 800 g of sediment without heavy metal addition. All treatments and controls were prepared in biological duplicate. In this study, the water-only group was designated as *Water* (W), the water sample from “water with sediment” group as *Water with sediment* (WS), and the sediment sample from “water with sediment” group as *Sediment* (S). The experiment lasted for 6 days. While W and WS samples were collected on days 0, 3, and 6, S samples were collected on days 0 and 6 to minimize disturbance.

### Analysis of physicochemical parameters

Environmental factors such as water temperature, pH, electrical conductivity (Cond), and dissolved oxygen (DO) were measured on-site using a water quality meter AQUACOMBO (Singapore). All measurements were taken immediately upon sample collection. Total nitrogen (TN) was quantified using the Spectroquant^®^ Nitrogen Cell Test, and total phosphorus (TP) was analyzed using the Phosphate Cell Test (Merck Millipore).

### DNA extraction, PCR amplification, and sequencing

For microbial community analysis, the collected water samples were filtered using a mixed cellulose membrane filter with a 0.22-µm pore size (Advantec MFS, Inc., Dublin, CA, USA). The filters were collected into the cryovials, and stored at −80 °C until DNA extraction. Genomic DNA was extracted using ChargeSwitch^®^ Forensic DNA Purification Kit (Thermo Fisher Scientific, Waltham, MA, USA). The DNA from sediment was extracted using FastDNA™ Spin DNA extraction kit (MP Biomedicals, USA), without any previous treatment. The bacterial 16 S rRNA gene for deep-sequencing was amplified using a universal primer set targeting the V3-V4 region, 341 F/805R (341 F: CCTACGGGNGGCWGCAG; 805R: GACTACHVGGGTATCTAATCC)^53^. The amplicons were purified using Agent AMPure XP beads (Beckman Coulter, Brea, CA, USA), and DNA was quantified with Quant-iT ds DNA HS Assay kit (Thermo Fisher Scientific). The paired-end sequencing of the purified amplicon was conducted by Macrogen Corporation (Seoul, South Korea) with Illumina MiSeq (Illumina, San Diego, CA, USA).

### Analysis of the microbial community

According to the standard operating procedure for amplicon sequence variant (ASV), the resulting raw sequences were processed using DADA2 (version 1.14.0)^54^. Different ASVs correspond to different genotypes. The sequences were aligned and classified using the Silva database v138^55^. ASVs identified as non-bacterial organisms, including Archaea and eukaryotic chloroplasts or mitochondria, as well as singletons, doubletons, and tripletons, were excluded from further analysis.

We estimated alpha diversity indices by calculating Chao1, evenness, and Shannon diversity indices using the “vegan” package in R^56^. To visualize the temporal change patterns of microbial community, we employed the “metaMDS” function within the “vegan” package in R to draw plots of non-metric multidimensional scaling (NMDS). The impact of potential physicochemical parameters on the microbial community was examined using distance-based redundancy analysis (dbRDA) implemented with the “capscale” function in the “vegan” package in R, which performs constrained analysis of principal coordinates (CAP) based on Bray-Curtis dissimilarity. The ordination results were visualized using the “ordiplot” function in the same package.

### Indicator species

To select indicator bacteria for As and Cd, indicator species analysis was conducted, using the “indicspecies” R package^57^. ASVs were considered valid indicators if they exhibited an indicator value greater than 0.5 and a *P* value less than 0.05, based on 999 permutation tests. The relative abundance of the selected indicator ASVs was extracted and standardized to z-scores across all samples to highlight treatment-specific patterns. For each ASV, z-scores were calculated as

$$\:\mathrm{z}=\frac{\left(\mathrm{x}-\:{\upmu\:}\right)}{\sigma\:}$$

where µ and σ represent the mean and standard deviation of the ASV, respectively. The standardized values were visualized using the “heatmap.2” function from the “gplots” R package without hierarchical clustering.

## Supplementary Information

Below is the link to the electronic supplementary material.

Supplementary Material 1

## Data Availability

The raw sequencing data have been deposited in the NCBI Sequence Read Archive (SRA) under BioProject accession number [PRJNA1293141].
