# Supplementary material for: Bacterial community shifts in response to arsenic and cadmium contamination in aquatic ecosystems: a microcosm study
Source: Sci Rep. 2026 Jun 9;16:26219. doi: 10.1038/s41598-026-57301-y (PMC13493909; doi:10.1038/s41598-026-57301-y)
Supplement: Supplementary file 1 [file 41598_2026_57301_MOESM1_ESM.docx]

**Supplementary materials**

**Bacterial community shifts in response to arsenic and cadmium contamination in aquatic ecosystems: a microcosm study**

**Hayoung Lee^1,2^, Mingyeong Kang^1,2^, Seonah Jeong^1^, Min-Seong Kim^1,2^, Ve Van Le^1^, So-Ra Ko^1^,** **Won-Suk Choi^1,2^, Dong-Yun Choi^1^, and Chi-Yong Ahn^1,2^***

^1^Cell Factory Research Center, Korea Research Institute of Bioscience and Biotechnology (KRIBB), 125 Gwahak-ro, Yuseong-gu, Daejeon 34141, Republic of Korea

^2^Department of Environmental Biotechnology, KRIBB School of Biotechnology, University of Science and Technology (UST), 217 Gajeong-ro, Yuseong-gu, Daejeon 34113, Republic of Korea

*Corresponding author

Chi-Yong Ahn

E-mail: cyahn@kribb.re.kr

Tel: +82-42-860-4329

Fax: +82-42-860-4594

**Supplementary material**

**Fig. S1.** Detailed comparative patterns of α-diversity indices: W (A), WS (B), and S (C). Each bar represents mean values, and error bars indicate standard deviation (SD) (*n* = 2).

**Table S1.** Physicochemical parameters measured at the beginning of the experiment. W and S denote water and sediment, respectively. All samples were measured in biological duplicates.

| **Day 0** | | | | |
| --- | --- | --- | --- | --- |
| **Sample** | **Temp. (^o^C)** | **DO (mg L^-1^)** | **pH** | **Cond. (µS cm^-1^)** |
| **W-1** | 20.8 | 6.6 | 7.40 | 437 |
| **W-2** | 21.1 | 7.1 | 7.41 | 437 |
| **WS-1** | 20.9 | 7.2 | 7.44 | 437 |
| **WS-2** | 21.0 | 6.8 | 7.41 | 439 |

**Table S2.** Physicochemical parameters measured on the day 3 of the experiment. W and S indicate water and sediment, respectively; number before the sample names (1 and 10) represent treated heavy metal concentrations; “C” represents the control, while “As” and “Cd” denote arsenic and cadmium treatments, respectively. All samples were measured in biological duplicates.

| **Day 3** | | | | |
| --- | --- | --- | --- | --- |
| **Sample** | **Temp. (^o^C)** | **DO (mg L^-1^)** | **pH** | **Cond. (µS cm^-1^)** |
| **WC-1** | 19.4 | 6.6 | 7.76 | 440 |
| **WC-2** | 23.0 | 7.4 | 8.58 | 446 |
| **1AsW-1** | 20.6 | 6.6 | 7.99 | 445 |
| **1AsW-2** | 22.9 | 6.4 | 8.27 | 449 |
| **10AsW-1** | 20.8 | 6.2 | 8.01 | 454 |
| **10AsW-2** | 22.8 | 6.0 | 8.12 | 455 |
| **1CdW-1** | 20.8 | 5.6 | 7.9 | 449 |
| **1CdW-2** | 22.5 | 5.5 | 7.91 | 451 |
| **10CdW-1** | 20.7 | 5.7 | 7.74 | 454 |
| **10CdW-2** | 22.9 | 5.4 | 7.93 | 458 |
| **WSC-1** | 20.9 | 10.9 | 8.50 | 418 |
| **WSC-2** | 23.3 | 9.8 | 9.01 | 416 |
| **10AsWS-1** | 21.5 | 8.7 | 8.30 | 445 |
| **10AsWS-2** | 22.8 | 9.3 | 8.20 | 444 |
| **10CdWS-1** | 21.7 | 5.8 | 7.95 | 455 |
| **10CdWS-2** | 22.9 | 5.6 | 7.73 | 447 |

**Table S3.** Physicochemical parameters measured on the day 6 of the experiment. W and S indicate water and sediment, respectively; number before the sample names (1 and 10) represent treated heavy metal concentrations; “C” represents the control, while “As” and “Cd” denote arsenic and cadmium treatments, respectively. All samples were measured in biological duplicates.

| **Day 6** | | | | |
| --- | --- | --- | --- | --- |
| **Sample** | **Temp. (^o^C)** | **DO (mg L^-1^)** | **pH** | **Cond. (µS cm^-1^)** |
| **WC-1** | 23.3 | 8.4 | 7.09 | 437 |
| **WC-2** | 25.8 | 6.9 | 7.36 | 454 |
| **1AsW-1** | 23.7 | 6.1 | 7.37 | 443 |
| **1AsW-2** | 25.6 | 5.5 | 7.39 | 460 |
| **10AsW-1** | 24.5 | 7.0 | 7.64 | 456 |
| **10AsW-2** | 25.6 | 4.8 | 7.53 | 460 |
| **1CdW-1** | 24.6 | 5.1 | 7.57 | 452 |
| **1CdW-2** | 24.9 | 4.6 | 7.52 | 454 |
| **10CdW-1** | 24.3 | 4.7 | 7.56 | 452 |
| **10CdW-2** | 25.3 | 4.8 | 7.49 | 470 |
| **WSC-1** | 24.8 | 10.0 | 8.96 | 364 |
| **WSC-2** | 29.6 | 9.0 | 9.26 | 385 |
| **10AsWS-1** | 25.7 | 10.6 | 9.45 | 412 |
| **10AsWS-2** | 26.1 | 11.7 | 9.52 | 421 |
| **10CdWS-1** | 25.6 | 6.5 | 8.72 | 450 |
| **10CdWS-2** | 26.1 | 5.5 | 8.37 | 458 |
| **SC-1** | 24.8 | 10.0 | 8.96 | 364 |
| **SC-2** | 29.6 | 9.0 | 9.26 | 385 |
| **10AsS-1** | 25.7 | 10.6 | 9.45 | 412 |
| **10AsS-2** | 26.1 | 11.7 | 9.52 | 421 |
| **10CdS-1** | 25.6 | 6.5 | 8.72 | 450 |
| **10CdS-2** | 26.1 | 5.5 | 8.37 | 458 |

**Table S4.** W-As indicators

|  | **stat^[1]^** | ***p*-value^[2]^** | **Phylum** | **Class** | **Order** | **Family** | **Genus** |
| --- | --- | --- | --- | --- | --- | --- | --- |
| ASV00443 | 0.974 | 0.0001^***^ | Bdellovibrionota | *Bdellovibrionia* | *Bdellovibrionales* | *Bdellovibrionaceae* | OM27 clade |
| ASV00451 | 0.935 | 0.0003^***^ | Bdellovibrionota | *Bdellovibrionia* | *Bacteriovoracales* | *Bacteriovoracaceae* | *Peredibacter* |
| ASV00503 | 0.935 | 0.0002^***^ | Proteobacteria | *Alphaproteobacteria* | *Rhizobiales* | *Beijerinckiaceae* | *alphaI cluster* |
| ASV00178 | 0.905 | 0.0008^***^ | Proteobacteria | *Alphaproteobacteria* | *Rhodobacterales* | *Rhodobacteraceae* | *Pseudorhodobacter* |
| ASV00387 | 0.898 | 0.0006^***^ | Bacteroidota | *Bacteroidia* | *Flavobacteriales* | *Flavobacteriaceae* | *Flavobacterium* |
| ASV00763 | 0.894 | 0.0007^***^ | Verrucomicrobiota | *Verrucomicrobiae* | *Verrucomicrobiales* | *Verrucomicrobiaceae* | NA |
| ASV00542 | 0.894 | 0.0007^***^ | Proteobacteria | *Alphaproteobacteria* | *Sphingomonadales* | *Sphingomonadaceae* | *Novosphingobium* |
| ASV00154 | 0.891 | 0.002^**^ | Bdellovibrionota | *Bdellovibrionia* | *Bacteriovoracales* | *Bacteriovoracaceae* | *Peredibacter* |
| ASV00289 | 0.879 | 0.0012^**^ | Proteobacteria | *Alphaproteobacteria* | *Sphingomonadales* | *Sphingomonadaceae* | *Porphyrobacter* |
| ASV00212 | 0.877 | 0.0048^**^ | Proteobacteria | *Gammaproteobacteria* | *Burkholderiales* | *Methylophilaceae* | *Methylotenera* |
| ASV00797 | 0.865 | 0.0012^**^ | Bacteroidota | *Bacteroidia* | *Flavobacteriales* | *Crocinitomicaceae* | *Fluviicola* |
| ASV00229 | 0.864 | 0.0041^**^ | Proteobacteria | *Alphaproteobacteria* | *Caulobacterales* | *Hyphomonadaceae* | *Hyphomonas* |
| ASV00750 | 0.864 | 0.0016^**^ | Verrucomicrobiota | *Verrucomicrobiae* | *Verrucomicrobiales* | DEV007 | NA |
| ASV00645 | 0.834 | 0.0039^**^ | Proteobacteria | *Alphaproteobacteria* | *Sphingomonadales* | *Sphingomonadaceae* | *Rhizorhapis* |
| ASV00333 | 0.826 | 0.0024^**^ | Proteobacteria | *Gammaproteobacteria* | *Burkholderiales* | *Alcaligenaceae* | GKS98 freshwater group |
| ASV00334 | 0.823 | 0.0028^**^ | Verrucomicrobiota | *Verrucomicrobiae* | *Verrucomicrobiales* | *Rubritaleaceae* | *Luteolibacter* |
| ASV01046 | 0.805 | 0.0054^**^ | Proteobacteria | *Gammaproteobacteria* | *Burkholderiales* | *Methylophilaceae* | *Methylotenera* |

**Table S5.** W-Cd indicators

|  | **stat** | ***p*-value** | **Phylum** | **Class** | **Order** | **Family** | **Genus** |
| --- | --- | --- | --- | --- | --- | --- | --- |
| ASV00072 | 1 | 0.0001^***^ | Bacteroidota | *Bacteroidia* | *Chitinophagales* | *Chitinophagaceae* | *Sediminibacterium* |
| ASV00148 | 1 | 0.0001^***^ | Proteobacteria | *Gammaproteobacteria* | *Burkholderiales* | *Methylophilaceae* | *Methylophilus* |
| ASV00372 | 1 | 0.0001^***^ | Proteobacteria | *Gammaproteobacteria* | *Burkholderiales* | *Comamonadaceae* | *Aquabacterium* |
| ASV00374 | 1 | 0.0001^***^ | Proteobacteria | *Gammaproteobacteria* | *Burkholderiales* | *Methylophilaceae* | *Methylophilus* |
| ASV00591 | 1 | 0.0001^***^ | Proteobacteria | *Gammaproteobacteria* | *Burkholderiales* | *Oxalobacteraceae* | NA |
| ASV00863 | 1 | 0.0001^***^ | Proteobacteria | *Gammaproteobacteria* | *Burkholderiales* | *Oxalobacteraceae* | NA |
| ASV01152 | 1 | 0.0001^***^ | Proteobacteria | *Gammaproteobacteria* | *Burkholderiales* | *Oxalobacteraceae* | NA |
| ASV00141 | 0.996 | 0.0001^***^ | Proteobacteria | *Gammaproteobacteria* | *Burkholderiales* | *Oxalobacteraceae* | *Undibacterium* |
| ASV00075 | 0.994 | 0.0001^***^ | Proteobacteria | *Gammaproteobacteria* | *Burkholderiales* | *Methylophilaceae* | *Methylophilus* |
| ASV00052 | 0.988 | 0.0001^***^ | Proteobacteria | *Gammaproteobacteria* | *Burkholderiales* | *Comamonadaceae* | *Curvibacter* |
| ASV00008 | 0.986 | 0.0060^**^ | Bacteroidota | *Bacteroidia* | *Chitinophagales* | *Chitinophagaceae* | *Sediminibacterium* |
| ASV00021 | 0.979 | 0.0054^**^ | Proteobacteria | *Gammaproteobacteria* | *Burkholderiales* | *Comamonadaceae* | *Limnohabitans* |
| ASV00201 | 0.972 | 0.0001^***^ | Proteobacteria | *Gammaproteobacteria* | *Burkholderiales* | *Oxalobacteraceae* | NA |
| ASV00394 | 0.95 | 0.0001^***^ | Proteobacteria | *Gammaproteobacteria* | *Burkholderiales* | *Comamonadaceae* | *Limnohabitans* |
| ASV00369 | 0.935 | 0.0003^***^ | Bacteroidota | *Bacteroidia* | *Chitinophagales* | *Chitinophagaceae* | *Sediminibacterium* |
| ASV00860 | 0.935 | 0.0004^***^ | Proteobacteria | *Gammaproteobacteria* | *Burkholderiales* | *Comamonadaceae* | *Limnohabitans* |
| ASV01205 | 0.935 | 0.0003^***^ | Bacteroidota | *Bacteroidia* | *Chitinophagales* | *Chitinophagaceae* | *Sediminibacterium* |
| ASV00009 | 0.929 | 0.0049^**^ | Bacteroidota | *Bacteroidia* | *Flavobacteriales* | *Flavobacteriaceae* | *Flavobacterium* |
| ASV00940 | 0.871 | 0.0012^**^ | Proteobacteria | *Gammaproteobacteria* | *Burkholderiales* | *Burkholderiaceae* | *Polynucleobacter* |
| ASV00742 | 0.836 | 0.0016^**^ | Proteobacteria | *Gammaproteobacteria* | *Burkholderiales* | *Comamonadaceae* | *Ramlibacter* |

**Table S6.** WS-As indicators

|  | **stat** | ***p*-value** | **Phylum** | **Class** | **Order** | **Family** | **Genus** |
| --- | --- | --- | --- | --- | --- | --- | --- |
| ASV01335 | 1 | 0.0025^**^ | Proteobacteria | *Alphaproteobacteria* | *Caulobacterales* | *Caulobacteraceae* | *Caulobacter* |
| ASV01608 | 1 | 0.0025^**^ | Proteobacteria | *Gammaproteobacteria* | *Burkholderiales* | *Comamonadaceae* | *Ottowia* |
| ASV00022 | 0.966 | 0.0203^*^ | Bacteroidota | *Bacteroidia* | *Cytophagales* | *Spirosomaceae* | *Fluviimonas* |
| ASV02651 | 0.952 | 0.0055^**^ | Bacteroidota | *Bacteroidia* | *Cytophagales* | *Microscillaceae* | NA |
| ASV00116 | 0.927 | 0.0304^*^ | Bacteroidota | *Bacteroidia* | *Cytophagales* | *Spirosomaceae* | *Lacihabitans* |
| ASV00079 | 0.91 | 0.0293^*^ | Proteobacteria | *Alphaproteobacteria* | *Sphingomonadales* | *Sphingomonadaceae* | *Rhizorhapis* |
| ASV00212 | 0.866 | 0.0225^*^ | Proteobacteria | *Gammaproteobacteria* | *Burkholderiales* | *Methylophilaceae* | *Methylotenera* |
| ASV00567 | 0.866 | 0.0218^*^ | Actinobacteriota | *Actinobacteria* | *Micrococcales* | *Microbacteriaceae* | NA |
| ASV01533 | 0.866 | 0.0218^*^ | Proteobacteria | *Alphaproteobacteria* | *Paracaedibacterales* | *Paracaedibacteraceae* | NA |
| ASV02584 | 0.866 | 0.0225^*^ | Bacteroidota | *Bacteroidia* | *Flavobacteriales* | *Flavobacteriaceae* | *Flavobacterium* |
| ASV02621 | 0.866 | 0.0225^*^ | Proteobacteria | *Gammaproteobacteria* | *Pseudomonadales* | *Pseudomonadaceae* | *Pseudomonas* |

**Table S7.** WS-Cd indicators

|  | **stat** | ***p*-value** | **Phylum** | **Class** | **Order** | **Family** | **Genus** |
| --- | --- | --- | --- | --- | --- | --- | --- |
| ASV00043 | 1 | 0.0016^**^ | Proteobacteria | *Gammaproteobacteria* | *Burkholderiales* | *Comamonadaceae* | *Limnohabitans* |
| ASV00051 | 1 | 0.0016^**^ | Bacteroidota | *Bacteroidia* | *Cytophagales* | *Spirosomaceae* | *Flectobacillus* |
| ASV00141 | 1 | 0.0016^**^ | Proteobacteria | *Gammaproteobacteria* | *Burkholderiales* | *Oxalobacteraceae* | *Undibacterium* |
| ASV00545 | 1 | 0.0016^**^ | Actinobacteriota | *Actinobacteria* | *Frankiales* | *Sporichthyaceae* | hgcI clade |
| ASV01058 | 1 | 0.0016^**^ | Proteobacteria | *Gammaproteobacteria* | *Burkholderiales* | *Comamonadaceae* | *Paucibacter* |
| ASV02270 | 1 | 0.0016^**^ | Proteobacteria | *Gammaproteobacteria* | *Burkholderiales* | *Comamonadaceae* | *Paucibacter* |
| ASV00075 | 0.965 | 0.0016^**^ | Proteobacteria | *Gammaproteobacteria* | *Burkholderiales* | *Methylophilaceae* | *Methylophilus* |
| ASV01434 | 0.932 | 0.001^***^ | Bacteroidota | *Bacteroidia* | *Flavobacteriales* | *NS9 marine group* | NA |
| ASV01518 | 0.913 | 0.0064^**^ | Actinobacteriota | *Acidimicrobiia* | *Microtrichales* | *Ilumatobacteraceae* | CL500-29 marine group |
| ASV00419 | 0.888 | 0.0112^*^ | Proteobacteria | *Gammaproteobacteria* | *Burkholderiales* | *Oxalobacteraceae* | *Undibacterium* |
| ASV02305 | 0.868 | 0.0138^*^ | Planctomycetota | *Planctomycetes* | *Pirellulales* | *Pirellulaceae* | *Rhodopirellula* |
| ASV00255 | 0.866 | 0.0224^*^ | Proteobacteria | *Gammaproteobacteria* | *Burkholderiales* | *Comamonadaceae* | *Ramlibacter* |
| ASV00702 | 0.866 | 0.0218^*^ | Proteobacteria | *Gammaproteobacteria* | *Burkholderiales* | *Burkholderiaceae* | *Polynucleobacter* |
| ASV02269 | 0.866 | 0.0218^*^ | Proteobacteria | *Gammaproteobacteria* | *Legionellales* | *Legionellaceae* | *Legionella* |
| ASV04115 | 0.866 | 0.0218^*^ | Proteobacteria | *Gammaproteobacteria* | *Burkholderiales* | *Methylophilaceae* | NA |
| ASV00052 | 0.832 | 0.019^*^ | Proteobacteria | *Gammaproteobacteria* | *Burkholderiales* | *Comamonadaceae* | *Curvibacter* |

**Table S8.** S-As indicators

|  | **stat** | ***p*-value** | **Phylum** | **Class** | **Order** | **Family** | **Genus** |
| --- | --- | --- | --- | --- | --- | --- | --- |
| ASV05687 | 0.985 | 0.0458^*^ | Bacteroidota | *Bacteroidia* | *Sphingobacteriales* | AKYH767 | NA |
| ASV04747 | 0.962 | 0.0486^*^ | Chloroflexi | *Anaerolineae* | *Caldilineales* | *Caldilineaceae* | *Litorilinea* |
| ASV00584 | 0.937 | 0.0458^*^ | Bacteroidota | *Bacteroidia* | *Flavobacteriales* | *Flavobacteriaceae* | *Flavobacterium* |
| ASV03436 | 0.935 | 0.0486^*^ | Acidobacteriota | *Vicinamibacteria* | *Vicinamibacterales* | *Vicinamibacteraceae* | NA |
| ASV01908 | 0.912 | 0.0486^*^ | Bacteroidota | *Bacteroidia* | *Cytophagales* | NA | NA |
| ASV02938 | 0.908 | 0.0486^*^ | Bacteroidota | *Bacteroidia* | *Bacteroidales* | *Bacteroidetes* vadinHA17 | NA |
| ASV01754 | 0.897 | 0.0486^*^ | Planctomycetota | *Phycisphaerae* | *Phycisphaerales* | *Phycisphaeraceae* | CL500-3 |

**Table S9.** S-Cd indicators

|  | **stat** | ***p*-value** | **Phylum** | **Class** | **Order** | **Family** | **Genus** |
| --- | --- | --- | --- | --- | --- | --- | --- |
| ASV03830 | 0.987 | 0.0496^*^ | Proteobacteria | *Gammaproteobacteria* | *Xanthomonadales* | *Xanthomonadaceae* | *Thermomonas* |
| ASV03428 | 0.981 | 0.0492^*^ | Proteobacteria | *Gammaproteobacteria* | *Burkholderiales* | *Methylophilaceae* | NA |
| ASV01951 | 0.977 | 0.0484^*^ | Bacteroidota | *Bacteroidia* | *Sphingobacteriales* | env.OPS 17 | NA |
| ASV00563 | 0.975 | 0.0484^*^ | Proteobacteria | *Gammaproteobacteria* | *Burkholderiales* | *Comamonadaceae* | *Hydrogenophaga* |
| ASV03214 | 0.974 | 0.0496^*^ | Proteobacteria | *Gammaproteobacteria* | *Burkholderiales* | *Comamonadaceae* | *Aquabacterium* |
| ASV02628 | 0.958 | 0.0492^*^ | Proteobacteria | *Gammaproteobacteria* | *Burkholderiales* | *Comamonadaceae* | *Simplicispira* |
| ASV03138 | 0.949 | 0.0484^*^ | Desulfobacterota | *Desulfuromonadia* | *Desulfuromonadales* | MA-28-I98C | NA |
| ASV00419 | 0.935 | 0.0492^*^ | Proteobacteria | *Gammaproteobacteria* | *Burkholderiales* | *Oxalobacteraceae* | *Undibacterium* |
| ASV02623 | 0.923 | 0.0484^*^ | Chloroflexi | *Dehalococcoidia* | S085 | NA | NA |
| ASV03301 | 0.918 | 0.0491^*^ | Firmicutes | *Clostridia* | *Lachnospirales* | *Lachnospiraceae* | *Cellulosilyticum* |
| ASV00376 | 0.914 | 0.0484^*^ | Proteobacteria | *Gammaproteobacteria* | *Burkholderiales* | *Comamonadaceae* | *Inhella* |
| ASV04039 | 0.904 | 0.0492^*^ | Bacteroidota | *Bacteroidia* | *Chitinophagales* | *Chitinophagaceae* | *Dinghuibacter* |

**Table S10.** Physicochemical parameters measured at the sampling site from 2018 to 2023 based on data from the Water Environment Information System (https://water.nier.go.kr; data for 2019 and 2020 were not available).

|  | **2018.05** | **2021.05** | **2022.05** | **2023.05** |
| --- | --- | --- | --- | --- |
| Temperature [℃] | 19.6 | 19 | 20.4 | 19.3 |
| pH | 8.4 | 7.6 | 7.8 | 7.7 |
| DO [mg L^-1^] | 10.1 | 8.5 | 8.6 | 8.5 |
| BOD [mg L^-1^] | 1.8 | 1.6 | 1.6 | 1.6 |
| COD [mg L^-1^] | 6.4 | 6.3 | 6.1 | 6 |
| SS [mg L^-1^] | 8.9 | 4.2 | 4.2 | 5 |
| EC [μS cm^-1^] | 307 | 360 | 447 | 427 |
| T-N [mg L^-1^] | 2.897 | 2.93 | 2.91 | 3.224 |
| NO_3_^-^-N [mg L^-1^] | 2.284 | 2.127 | 2.293 | 2.535 |
| NH_3_-N [mg L^-1^] | 0.065 | 0.297 | 0.133 | 0.157 |
| T-P [mg L^-1^] | 0.026 | 0.038 | 0.034 | 0.044 |
| PO_4_^-^-P[mg L^-1^] | 0.001 | 0.016 | 0.008 | 0.021 |
| Chlorophyll-*a* [mg m^-3^] | 31.7 | 8.3 | 8.9 | 9.1 |
| TOC [mg L^-1^] | - | 4.4 | 4.1 | 4.4 |
| Phenols [mg L^-1^] | 0 | 0 | 0 | 0 |
| Cd [mg L^-1^] | 0 | 0 | 0 | 0 |
| CN^-^ [mg L^-1^] | 0 | 0 | 0 | 0 |
| Pb [mg L^-1^] | 0 | 0 | 0 | 0 |
| Cr^6+^ [mg L^-1^] | 0 | 0 | 0 | 0 |
| As [mg L^-1^] | 0 | 0 | 0 | 0 |
| Hg [mg L^-1^] | 0 | 0 | 0 | 0 |
| Alkylbenzene sulfonate [mg L^-1^] | 0 | 0 | 0 | 0 |

1. The stat value (Indicator Value, IndVal) represents the strength of association between a specific taxon and a group. It ranges from 0 to 1, where a higher value indicates that the taxon is both more abundant and more consistently present in that group.
2. Statistical significance is denoted as follows: *** for *P* < 0.001, ** for *P* < 0.01, * for *P* < 0.05.
